# Effect of Strontium Substitution on the Tribocatalytic Performance of Barium Titanate

**DOI:** 10.3390/ma16083160

**Published:** 2023-04-17

**Authors:** Siyu Liu, Yaodong Yang, Yongming Hu, Wei-Feng Rao

**Affiliations:** 1 Faculty of Mechanical Engineering, Shandong Institute of Mechanical Design and Research, Qilu University of Technology (Shandong Academy of Sciences), Jinan 250353, China; 2 Hubei Key Laboratory of Ferro- and Piezoelectric Materials and Devices, Faculty of Physics & Electronic Science, Hubei University, Wuhan 430062, China

**Keywords:** tribocatalysis, barium strontium titanate, substitution, friction, dye degradation

## Abstract

This study investigates the impact of Sr doping on the tribocatalytic performance of BaTiO_3_ in degrading organic pollutants. Ba_1-x_Sr_x_TiO_3_ (x = 0–0.3) nanopowders are synthesized and their tribocatalytic performance evaluated. By doping Sr into BaTiO_3_, the tribocatalytic performance was enhanced, resulting in an approximately 35% improvement in the degradation efficiency of Rhodamine B using Ba_0.8_Sr_0.2_TiO_3_. Factors such as the friction contact area, stirring speed, and materials of the friction pairs also influenced the dye degradation. Electrochemical impedance spectroscopy revealed that Sr doping improved BaTiO_3_’s charge transfer efficiency, thereby boosting its tribocatalytic performance. These findings indicate potential applications for Ba_1-x_Sr_x_TiO_3_ in dye degradation processes.

## 1. Introduction

Tribocatalysis is a green catalytic process that can be applied in various areas such as wastewater degradation [1], nitrogen fixation [2], and combustible gas generation [3]. This process can collect mechanical energy and excite electron-hole pairs through friction between two materials. Tribocatalytic degradation of organic pollutants has been reported for Ba_4_Nd_2_Fe_2_Nb_8_O_30_ [4], ZnO [5], TiO_2_ [6], Bi_2_WO_6_ [7], etc.

One of the keys to improving the tribocatalytic performance is to speed up the transfer of electron-hole pairs to the material surface after friction. Current charge transfer enhancement methods include building composites or fabricating materials with high aspect ratios. For example, Hu et al. [8] prepared biochar-zinc oxide (BC-ZnO) composites and showed that an increase in the surface defect density of carbon materials leads to an increase in charge transfer efficiency, which in turn promotes the tribocatalytic performance. Yang et al. [9] also suggested that preparing CdS nanowires with high aspect ratios enables charge separation and carrier transfer during friction stirring. However, both approaches have some shortcomings. For example, preparing composites requires complex processes and carefully designing the interface between the two materials. In addition, the crystal structure often limits the growth of single crystal nanowires. Preparing nanocatalysts with high aspect ratios is hardly a universal strategy to enhance charge transfer efficiency. Therefore, finding a simple and effective method to enhance the charge transport efficiency is crucial to improve tribocatalytic performance.

Substitution may be a suitable method to modulate the charge transfer efficiency [10,11]. For example, Yu et al. [12] changed the conductivity of SrTiO_3_ by doping with ions, which improved its sonocatalytic performance. BaTiO_3_, the most widely studied ABO_3_ type perovskite ferroelectric material, has ferroelectric properties, and its internal electric field induced by spontaneous polarization can promote carrier separation [13,14,15]. In addition, the perovskite lattice of BaTiO_3_ can accommodate ions of different sizes while maintaining the good perovskite structure, allowing some doping elements to be localized in BaTiO_3_ [16,17]. In the tribocatalytic field, Yang et al. [18] reported that Ba_0.7_Sr_0.3_TiO_3_ showed tribocatalytic performance via substitution. Therefore, doping Sr in BaTiO_3_ matrix is an effective solution for improving performance. However, the present study on the tribocatalysis of barium strontium titanate is limited to an individual component, which cannot give out a complete picture of the effect on strontium doping. There are two key questions to be answered: How do the different doping amounts of Sr affect the tribocatalytic performance? Whether the improvement comes from the variation of charge transfer?

To obtain materials with different charge transport efficiency and exclude the interference of morphology, different Sr-doped BaTiO_3_ with similar morphology and particle size were prepared. The charge transport efficiency of Ba_1-x_Sr_x_TiO_3_ (x = 0–0.3) was analyzed by electrochemical impedance spectroscopy. The tribocatalytic degradation of organic dye Rhodamine B (RhB) by Ba_1-x_Sr_x_TiO_3_ (BST) under magnetic stirring was studied. These results elaborated on the relationship between A site substitution, charge transport efficiency, and tribocatalytic process. After finding the optimized amount of doping component, the effects of factors such as friction contact area, stirring speed, and materials of friction pairs on the tribocatalytic performance were investigated. In addition, the possible mechanism of tribocatalysis was explored, and the active species elimination experiment demonstrated that the active species produced in tribocatalysis degraded the dye. Doping BaTiO_3_ with an appropriate amount of Sr significantly improved tribocatalytic performance, stemming from enhanced charge transport efficiency. This advancement provides a novel approach for utilizing the widely distributed friction energy in the environment to treat wastewater.

## 2. Materials and Methods

### 2.1. Synthesis of BST

Ba_1-x_Sr_x_TiO_3_ or BST nanopowders were prepared by hydrothermal assisted sol-gel method [19], where x = 0, 0.1, 0.2, 0.3 (respectively expressed as BTO, BST-0.1, BST-0.2, BST-0.3). Barium acetate (99%, Aladdin), strontium acetate (≥99%, Adamas-beta), acetic acid (≥99.7%, Alfa Aesar), ethylene glycol monomethyl ether (99%, Innochem), tetrabutyl titanate (≥99%, Innochem) and hexadecyltrimethylammonium bromide (99%, Innochem) were used as starting materials. First of all, barium acetate and strontium acetate were dissolved in 20 mL of acetic acid at 60 °C and stirred until dissolved, which was recorded as solution A. At the same time, 3.52 mL of tetrabutyl titanate was added to 16 mL of ethylene glycol methyl ether, recorded as solution B. Solution B was added into solution A, and 0.1 g of hexadecyltrimethylammonium bromide was added to the mixed solution. Then, the solution was transferred to a stainless steel autoclave lined with Teflon and held at 150 °C for 6 h. After that, the mixture solution was stirred in a beaker at 60 °C for 3 h. Finally, the samples were annealed at 500 °C for 1 h. After grinding with mortar, the samples were annealed at 800 °C for 3 h.

### 2.2. Characterizations

Phase structure of BST was tested through X-ray diffraction (XRD, SmartLab SE, Tokyo, Japan) using Cu Kα radiation. Scanning electron microscopy (SEM, FEI QUANTA FEG250, Hillsboro, OR, USA) was used to analyze the morphology of nanopowders. Energy dispersive X-ray spectroscopy (EDS) was used to analyze the elemental composition. Raman spectroscopy (DXR Microscope, Thermo Fisher, Madison, WI, USA) was used to determine the structure of the BTO. The electrochemical impedance spectroscopy (EIS) was determined by an electrochemical workstation (CHI650E, Chenhua Instrument Company) with platinum as the counter electrode, Ag/AgCl as the reference electrode, glassy carbon electrode as the working electrode, and 0.5 mol/L Na_2_SO_4_ solution as the electrolyte. The test conditions for EIS were: an initial voltage of 0.366 V and a test frequency of 10^5^ Hz–0.1 Hz. The elements in the sample and binding energy states were analyzed using X-ray photoelectron spectroscopy (XPS, Escalab 250, St. Petersburg, FL, USA). The UV-Vis absorption spectrum of BST was measured by UV-Vis near infrared spectrophotometer (Agilent, Cary 7000, Santa Clara, CA, USA). The XRD patterns were analyzed by Rietveld refinement using FullProf Suite.

### 2.3. Tribocatalytic Dye Degradation

The tribocatalytic performance of BST was evaluated by decomposing RhB solution under regular magnetic stirring at 25 °C (in the dark, the schematic diagram of magnetic stirring is shown in Figure S1). In a typical experiment, 30 mg BST nanopowders were added to a flat-bottomed glass, followed by 30 mL of 5 mg/L RhB solution. Then, the solution was stirred at 300 rpm with a polytetrafluoroethylene (PTFE) magnetic bar (Φ8 mm × 30 mm). At regular intervals, 3 mL RhB solution was taken and filtered. The absorption spectrum of RhB solution at 554 nm was recorded by a UV-Vis spectrophotometer (UV2800S spectrophotometer, SOPTOP, China) to evaluate the concentration change.

Before the catalytic reaction, all solutions (with or without catalysts) were allowed to stand for 30 min and without stirring to reach adsorption/desorption equilibrium. All tribocatalysis experiments were performed under dark conditions.

### 2.4. Detection of Active Species

To further investigate the tribocatalytic mechanism of BST, free radicals and hole trapping agents were used to detect the active species in the tribocatalytic degradation process. Experiments were designed by adding 1 mM tert-butanol (TBA), benzoquinone (BQ), and ethylenediaminetetraacetic acid disodium (EDTA-2Na) to a 30 mL RhB solution containing the catalysts. Active species detection experiments were performed at 300 rpm via a magnetic bar. RhB solution was taken every 2 h and measured by a UV-Vis spectrophotometer.

## 3. Results and Discussion

### 3.1. Structure and Morphology

**Figure 1:**
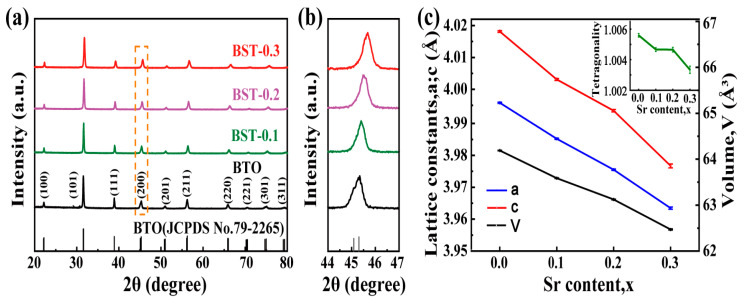
XRD patterns of Ba_1-x_Sr_x_TiO_3_ (0 ≤ x ≤ 0.3) nanopowders in the 2θ ranges of (**a**) 20° to 80° and (**b**) 44° to 47°; (**c**) evolution of lattice constants (**a**,**c**), cell volume (V), and tetragonality (c/a, insert) with different Sr contents.

Figure 1a is a typical XRD pattern of the BST, which shows that Sr is doped into the tetragonal perovskite structure of BTO (JCPDS No.79-2265) with good crystallization and no other impurity. Figure 1b shows the amplified (200) peak at 2θ = 44°–47°. As the small radius Sr^2+^ replaces the large radius Ba^2+^, the diffraction peaks shift to higher angles and the lattice constants decrease with adding Sr [12,20]. The lattice constants of the BST nanopowders were obtained by refining the XRD patterns using the Rietveld method. Prepared BST nanopowders are tetragonal structures with spontaneous polarization at room temperature, and their lattice constants and tetragonality (c/a value) decrease with increasing Sr, which is consistent with other literature [21] (see Figure 1c). To further prove the BTO had a tetragonal structure, Raman spectroscopy was performed (Figure S2). The characteristic peaks of BTO appear around 263 cm^−1^ [A_1_(TO)], 308 cm^−1^ [E(TO + LO),B_1_], 524 cm^−1^ [E(TO),A_1_(TO)], 716 cm^−1^ [E(LO),A_1_(LO)] [22,23], respectively. Among them, the characteristic peaks at 308 cm^−1^ and 716 cm^−1^ are the characteristic peaks of the tetragonal structure of BTO [24,25].

**Figure 2:**
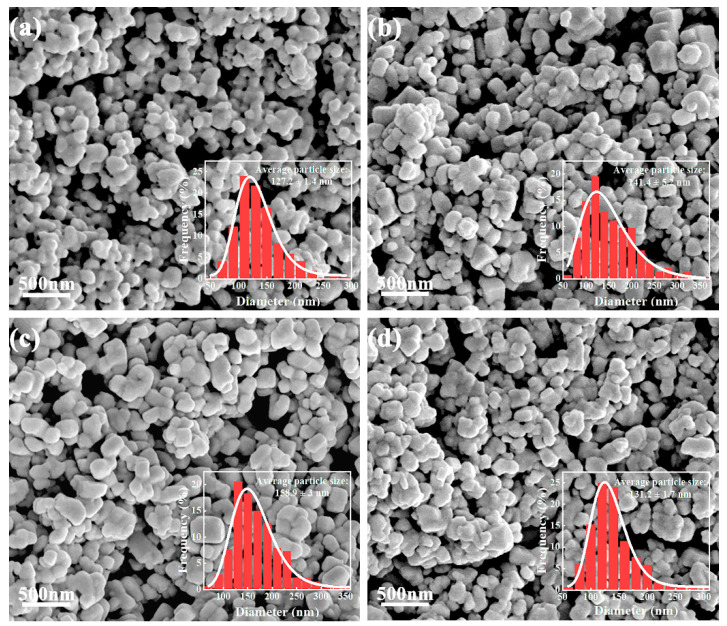
SEM images of Ba_1-x_Sr_x_TiO_3_ nanopowders with different x: (**a**) x = 0; (**b**) x = 0.1; (**c**) x = 0.2; (**d**) x = 0.3.

SEM images (Figure 2) show that all BST nanopowders have typical particle morphology, and the average particle sizes of BTO, BST-0.1, BST-0.2, and BST-0.3 nanopowders are 127 nm, 141 nm, 159 nm, and 131 nm in that order. With the increase of strontium doping, the particle size of BST nanopowders first increases and then decreases, reaching a maximum at BST-0.2. Elemental analysis shows that the content ratios of Ba, Sr, and Ti are very close to the designed stoichiometric ratios from Figure S3. 

**Figure 3:**
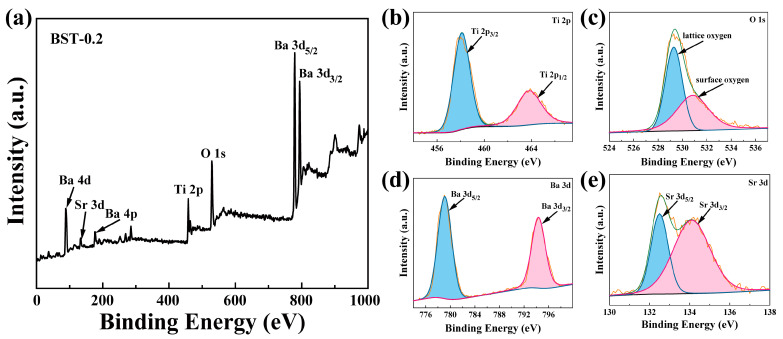
XPS spectra of BST-0.2 nanopowders: (**a**) XPS survey spectrum; (**b**) Ti 2p; (**c**) O 1s; (**d**) Ba 3d; (**e**) Sr 3d.

The elemental species and binding energy states of the BST-0.2 nanopowders were analyzed using XPS, as shown in Figure 3. Figure 3a shows the survey spectra of BST-0.2, indicating the presence of Ba, Sr, Ti, and O elements. Figure 3b shows the high-resolution XPS spectra of Ti 2p with binding energies at 458.1 eV and 463.8 eV corresponding to Ti 2p_3/2_ and Ti 2p_1/2_ of Ti^2+^, respectively. In Figure 3c, the binding energies at 529.3 eV and 530.9 eV correspond to lattice oxygen and surface adsorption oxygen [18], respectively. The binding energies of Ba 3d_5/2_ and Ba 3d_3/2_ have peak binding energies of 779.1 eV and 794.4 eV, respectively (Figure 3d). In Figure 3e, the binding energies at 132.5 eV and 134.1 eV correspond to Sr 3d_5/2_ and Sr 3d_3/2_, respectively. The binding energy state of BST-0.2 is essentially the same as previously reported [12], indicating that BST-0.2 nanopowders have been successfully synthesized.

### 3.2. Tribocatalytic Activity

**Figure 4:**
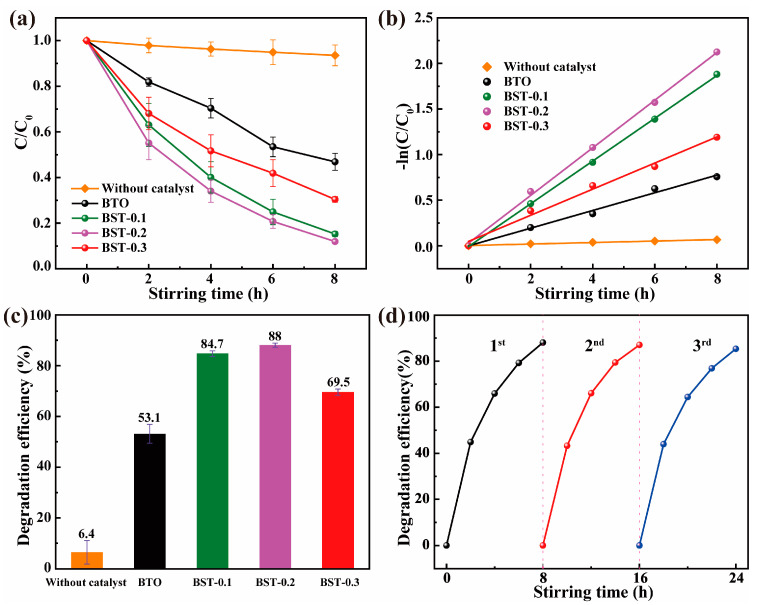
(**a**) Tribocatalytic degradation performance; (**b**) kinetic curves; (**c**) degradation efficiency of BST nanopowders for RhB solution; (**d**) degradation efficiency of BST-0.2 nanopowders after three cycles.

The tribocatalytic performance of the BST nanopowders was demonstrated by degrading RhB solution. Figure 4a shows the tribocatalytic performance of BST nanopowders measured under the same conditions, and the results indicate that BTO doped with Sr improves the dye degradation, where the degradation efficiencies of BTO, BST-0.1, BST-0.2, and BST-0.3 are 53.1%, 84.7%, 88% and 69.5% (reaction for 8 h), respectively. In addition, the low degradation efficiency of tribocatalysis in the absence of the catalyst shows the vital role of the ferroelectric catalyst in this process. Subsequently, we fitted the catalytic data via the pseudo-first-order kinetics mode:

(1) $$\ln\left(\frac{C}{C_{0}}\right)=-k\;\cdot \;t$$

where k represents the rate constant and t is reaction time. The k values for the BTO and BST-0.2 nanopowders in Figure 4b were 0.0971 h^−1^ and 0.2613 h^−1^, respectively. The latter one was about 2.7 times higher than pure BTO. In Figure 4c, with the increase of strontium content, the dye degradation efficiency showed a trend of increasing and then decreasing, which indicated adding Sr would affect tribocatalytic degradation remarkably. In addition, the adsorption of RhB solution by BST nanopowders was also investigated. As shown in Figure S4, the adsorption efficiency of BST nanopowders on dyes depended on the particle size. Still, the contribution to the final dye degradation was insignificant due to the slight difference in the adsorption efficiency of BST nanopowders on dyes.

Then recycling measurements of BST-0.2 were shown in Figure 4d. After three cycles, the dye degradation efficiency did not decrease significantly. The XRD examination of the recovered BST-0.2 nanopowders revealed that the positions of the characteristic peaks did not change (Figure S5), indicating that the prepared nanopowders have high stability and reusability.

**Figure 5:**
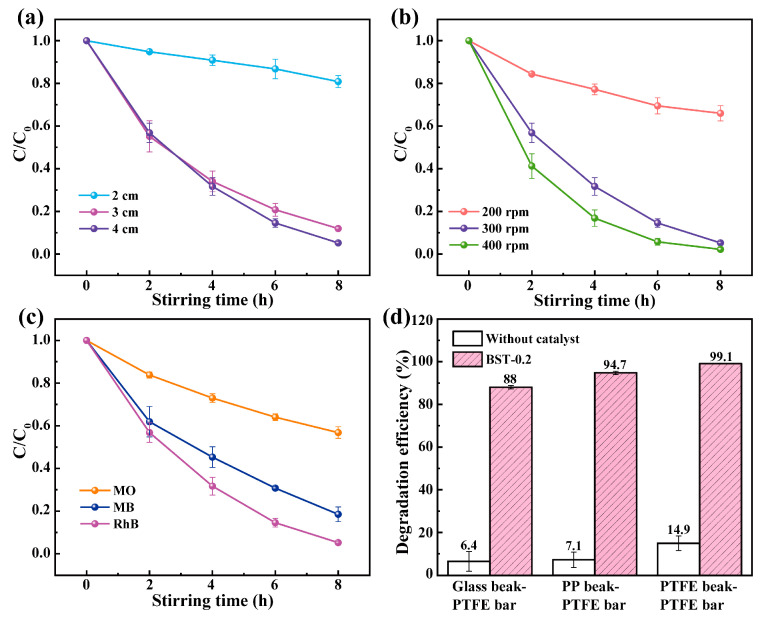
Effect of experimental factors on the tribocatalytic performance of BST-0.2 nanopowders: (**a**) length of the magnetic bar; (**b**) stirring speed; (**c**) type of dye; (**d**) materials of the friction pairs.

Since BST-0.2 nanopowders exhibit excellent tribocatalytic performance, the effects of a series of factors such as friction contact area, stirring speed, type of dye, and materials of the friction pairs were investigated by using BST-0.2 nanopowders as catalysts. The increase of the frictional contact area between the magnetic bar and the beaker was achieved by increasing the length of the magnetic bar. As shown in Figure 5a, with the increase of the length of the magnetic bar, more catalysts were subjected to the frictional action of the magnetic bar and beaker and involved in the degradation of RhB solution, thus improving the tribocatalytic performance. Next, the effect of stirring speed on the tribocatalytic performance was investigated under the action of a 4 cm long magnetic bar. As shown in Figure 5b, the degradation efficiency of RhB solution was low at the stirring speed of 200 rpm. As the stirring speed increased to 400 rpm, the friction between the catalyst and the magnetic bar increased, which improved the tribocatalytic performance. Therefore, increasing friction contact area and stirring speed positively affect the improvement of tribocatalytic performance.

The reliability of degradation of organic dyes was studied at a stirring speed of 300 rpm with a 4 cm magnetic bar. As shown in Figure 5c, RhB, Methylene blue (MB), and Methyl orange (MO) solutions were degraded under the same conditions. Compared with MO and MB solutions, BST-0.2 catalyst has a more robust catalytic degradation performance for RhB solution, which indicates that RhB solution is the most easily degraded. Different dyes show different tribocatalytic performance because different molecular structures of dyes require different energy to break chemical bonds [26]. The dyes degrade easily for a RhB solution containing low bond energy C-N or N-N single bonds. However, if the dye contains C=N or N=N double bonds, it often needs to provide higher energy to break these chemical bonds. Hence, MO and MB solutions are relatively difficult to degrade.

The effect of the materials of the friction pairs (composed of magnetic bar and beaker) on the tribocatalytic performance was studied by changing the materials of the beakers under the influence of a magnetic bar with a stirring speed of 300 rpm and a length of 3 cm. As shown in Figure 5d, the degradation efficiency of RhB solution is not high under agitation without a catalyst. However, with the addition of the BST-0.2 catalyst, the degradation efficiency was greatly improved. After 8 h degradation, the degradation efficiencies of the RhB solution were 88%, 94.7%, and 99.1% in the friction pairs of Glass beaker-PTFE bar, PP beaker-PTFE bar, and PTFE beaker-PTFE bar, respectively. The friction process involves the transfer of friction charge [27], and the charge transfer generated by friction between different materials varies depending on the electron affinity of the materials [28]. According to the triboelectric sequence table [29], the material at the bottom of the table is more likely to gain electrons when rubbed against the catalyst. PTFE can absorb more electrons, so BST-0.2 catalyst releases more electrons when in contact with PTFE beaker and PTFE bar, which gives it a very high tribocatalytic efficiency [30]. BST-0.2 nanopowders had higher dye degradation potential than other reported data (Table S1) because of Sr doping in BTO and different friction pair materials.

### 3.3. Photoelectric Properties

**Figure 6:**
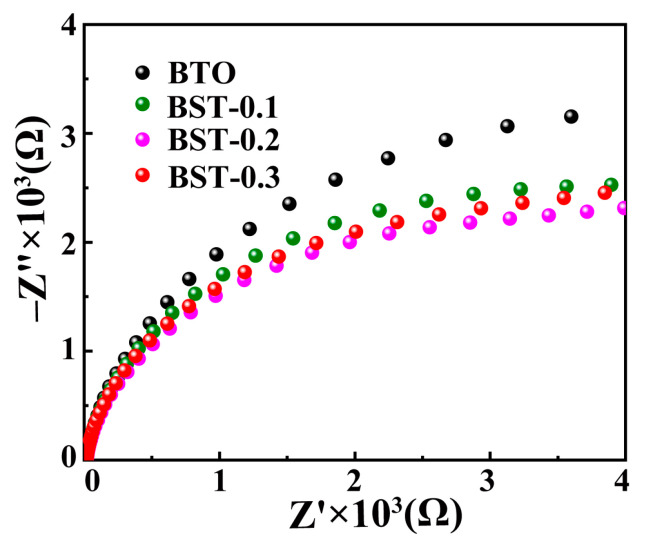
Electrochemical impedance spectroscopy of BST nanopowders.

To further investigate the mechanism for Sr doping to improve the tribocatalytic performance of BTO, the charge transfer efficiency of the BST catalyst was analyzed using electrochemical impedance spectroscopy (EIS). The smaller the radius of the arc, the lower the resistance of charge transfer and the higher the efficiency of charge transfer [31]. As shown in Figure 6, the doping of Sr in the BTO reduces the charge transfer resistance in all cases. Among them, the BST-0.2 nanopowder has the smallest arc radius, corresponding to a minor charge transfer resistance; the BTO nanopowder has the largest arc radius, indicating that the charge transfer is severely hindered. 

In addition, the reason why BST-0.2 exhibits the best tribocatalytic performance may be related to the fact that more friction energy is collected during the friction process. The collection of friction energy is related to the band gap width of the catalyst, and a narrow band gap width indicates that less energy is required to generate carriers, which would facilitate the collection of friction energy [9]. As seen in Figure S6, as the band gap width of BST becomes narrower with increasing strontium doping, the BST-0.2 nanopowders may collect more energy during friction.

### 3.4. Tribocatalytic Mechanism

**Figure 7:**
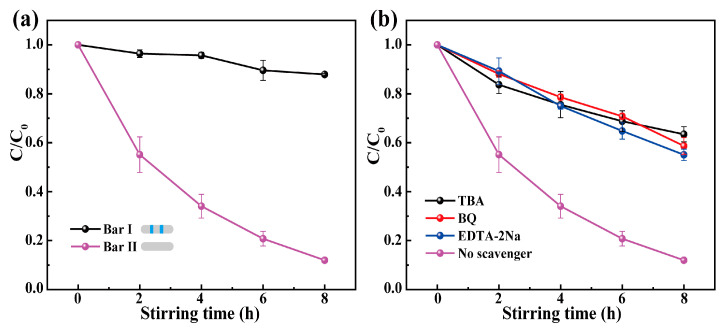
(**a**) Tribocatalytic degradation performance of BST-0.2 nanopowders for RhB solution under the conditions of contact or incomplete contact between the magnetic bar and the bottom of the beaker (modified magnetic bar with two rubber rings, noted as Bar I); (**b**) Effect of scavengers on the tribocatalytic performance of BST-0.2 nanopowders.

According to previous studies, the frictional interaction between materials can produce electron transfer and then generate active substances [4,27]. That is why the degradation can still occur in the absence of the catalyst. However, it should be noted that in Figure 4a, the degradation without the catalyst was poor, indicating that the degradation effect due to frictional interaction between two friction pairs is limited. To better understand the tribocatalysis, the effect of the frictional interaction between the catalyst and the PTFE magnetic bar on the tribocatalytic performance was investigated. As shown in Figure 7a, adding two rubber rings to the 3 cm long magnetic bar (noted as Bar I) reduces the frictional interaction between the catalyst and the magnetic bar. At 300 rpm, the degradation efficiency of the RhB solution by the Bar I was much worse than that of the unmodified magnetic bar. The results indicate that in the presence of the catalyst, the catalyst can effectively degrade the dye solution only by the frictional action between the magnetic bar and the bottom of the beaker, which reflects the importance of friction in dye degradation.

To investigate the role of active species in the tribocatalytic process, an active species elimination experiment was performed by adding scavengers: TBA (a hydroxyl radical scavenger), BQ (a superoxide radical scavenger), and EDTA-2Na (a hole scavenger) [32]. In Figure 7b, the degradation of RhB solution was inhibited after adding the scavengers, and the active species that played a role in the degradation were •OH, •O_2_^−^, and h^+^ in order of importance.

**Figure 8:**
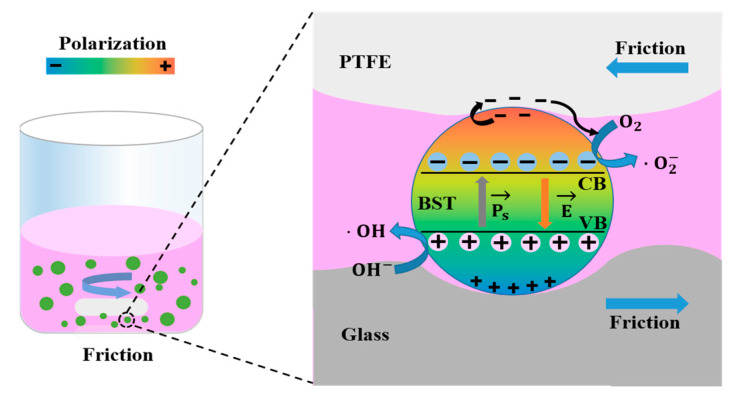
Mechanism diagram of tribocatalysis of BST nanopowders.

Based on our results, the mechanism of the tribocatalytic phenomenon from ferroelectric materials may arise from frictional charge transfer and the action of the internal electric field. As ferroelectric materials, the dipole moment is generated due to the non-coincidence of positive and negative charge centers, which produces spontaneous polarization [33,34]. Under normal conditions, surface charges induced by spontaneous polarization are compensated due to screening behaviors (neutralized by internal defect or molecules from outside) [18,35]. The mechanism diagram of tribocatalysis of BST nanopowders is shown in Figure 8. During stirring, the PTFE magnetic bar exerts pressure on the beaker, and the catalyst is temporarily immobilized at the contact interface between the PTFE magnetic bar and the beaker. The pressure forces a strong electron cloud overlap between the BST nanopowders and the magnetic bar [27], which transfers electrons from the catalyst to the PTFE magnetic bar. These charges induce redox reactions at the interface between the catalyst and the friction pairs. In this process, the materials of the friction pairs affect the friction charge transfer. At the same time, the catalyst was subjected to cyclic frictional forces between the magnetic bar and the beaker due to the frictional stirring effect of the PTFE magnetic bar. When the catalyst is fixed on the contact interface between the PTFE magnetic bar and the beaker, the frictional charge transfer on the material surface breaks the screening behaviors of the ferroelectric material. At this time, the internal electric field of the material induces electrons to jump from the valence band to the conduction band. The separated electrons and holes are transferred to the material surface. The electrons and holes on the material surface undergo redox reactions with O_2_ and OH^-^ in the solution to generate active species, which degrade the dye [36]. During this process, the increase of charge transfer efficiency is beneficial to accelerate the degradation efficiency of dyes.

The process can be summarized as the following reaction:

(2) $$\mathrm{BST}\;\overset{\mathrm{Triboelectric}\;\mathrm{energy}}{\to }{\;\mathrm{BST}+h}_{\mathrm{VB}}^{+}{+e}_{\mathrm{CB}}^{-}$$

(3) $${\mathrm{OH}}^{-}{+h}_{\mathrm{VB}}^{+}\;\to \;•\mathrm{OH}$$

(4) $$O_{2}{+e}_{\mathrm{CB}}^{-}\;\to {\;•O}_{2}^{-}$$

(5) $${\mathrm{Dye}+•\mathrm{OH}\;(•O}_{2}^{-})\;\to \;\mathrm{Degradation}$$

where $h_{\mathrm{VB}}^{+}$, $e_{\mathrm{CB}}^{-}$ represent the holes and electrons excited by tribocatalytic process, respectively.

## 4. Conclusions

In summary, Ba_1-x_Sr_x_TiO_3_ nanopowders were prepared by a hydrothermal assisted sol-gel method, and their tribocatalytic performance was investigated after the A site substitution. Increasing Sr^2+^, the tribocatalytic performance firstly increased and then decreased. Ba_0.8_Sr_0.2_TiO_3_ showed the best tribocatalytic performance, 88% RhB was degraded in 8 h, while undoped BaTiO_3_ degraded 53.1% only. EIS data indicated that the charge transfer affected the tribocatalytic performance directly. The improvement of tribocatalytic performance by changing the materials of the friction pairs was significant too. The degradation efficiency of RhB solution by the friction pairs consisting of PTFE beaker-PTFE bar reached 99.1% in 8 h. The active species experiments showed that the BST could generate three main active species during friction. This work clearly shows that BST nanopowders can harvest friction energy to be used in the dye wastewater treatment.

## Data Availability

All data is included in the manuscript.

## Supplementary Materials

The following supporting information can be downloaded at: https://www.mdpi.com/article/10.3390/ma16083160/s1, Figure S1: Schematic diagrams of a regular magnetic stirring setup; Figure S2: Raman spectra of BTO nanopowders; Figure S3: EDS patterns of Ba_1−x_Sr_x_TiO_3_ nanopowders when x is: (a) x = 0; (b) x = 0.1; (c) x = 0.2; (d) x = 0.3; Figure S4: Adsorption efficiency of BST nanopowders on RhB solution within 30 min; Figure S5: XRD patterns of BST-0.2 nanopowders before and after three cycles; Figure S6: UV-Vis absorption spectrum of BST nanopowders; Table S1: Comparison of the tribocatalytic performance with various materials [5,18,35,37,38].
