# Supplementary material for: Effect of Strontium Substitution on the Tribocatalytic Performance of Barium Titanate
Source: Materials (Basel). 2023 Apr 17;16(8):3160. doi: 10.3390/ma16083160 (PMC10143700; doi:10.3390/ma16083160)
Supplement: Supplementary file 1 [file materials-16-03160-s001.zip › materials-2332996-supplementary.pdf]

Supplementary Materials

Effect of strontium substitution on the tribocatalytic performance of barium titanate

Siyu Liu [1], Yaodong Yang [1,*], Yongming Hu [2] and Wei-Feng Rao [1,*]

1 Faculty of Mechanical Engineering, Shandong Institute of Mechanical Design and Research, Qilu University of Technology (Shandong Academy of Sciences), Jinan 250353, China

2 Hubei Key Laboratory of Ferro- and Piezoelectric Materials and Devices, Faculty of Physics & Electronic Science, Hubei University, Wuhan 430062, China

* Correspondence: yaodongy@qlu.edu.cn (Y.Y.); wfrao@qlu.edu.cn (W.-F.R.)

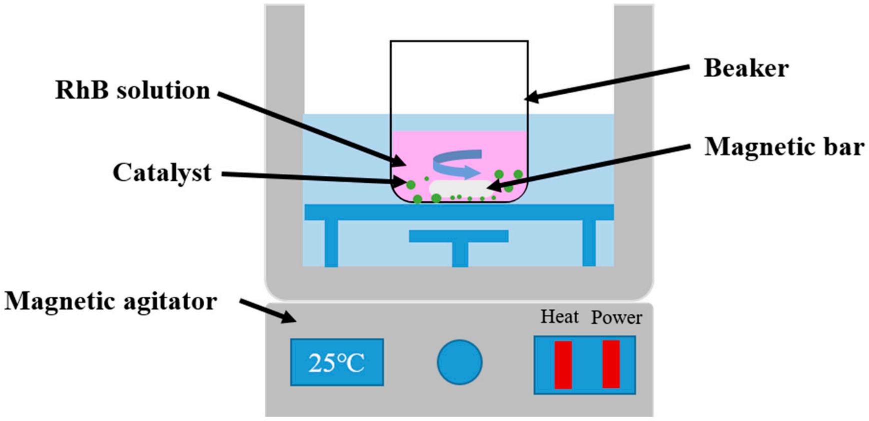

Figure S1. Schematic diagrams of a regular magnetic stirring setup.

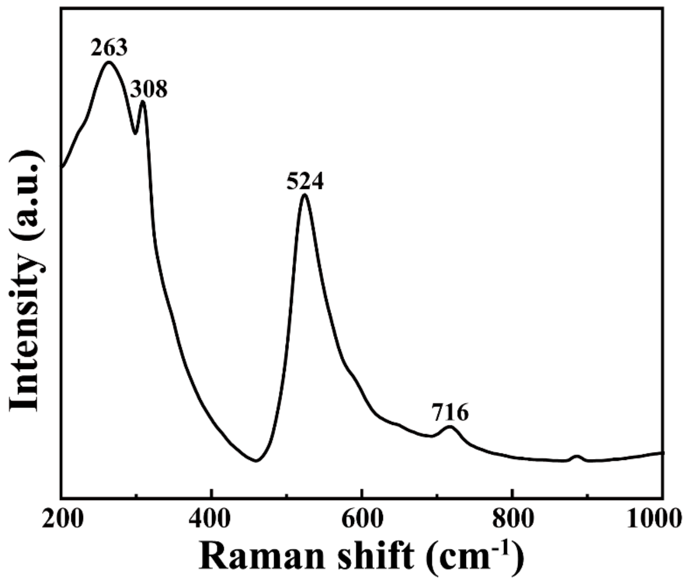

Figure S2. Raman spectra of BTO nanopowders.

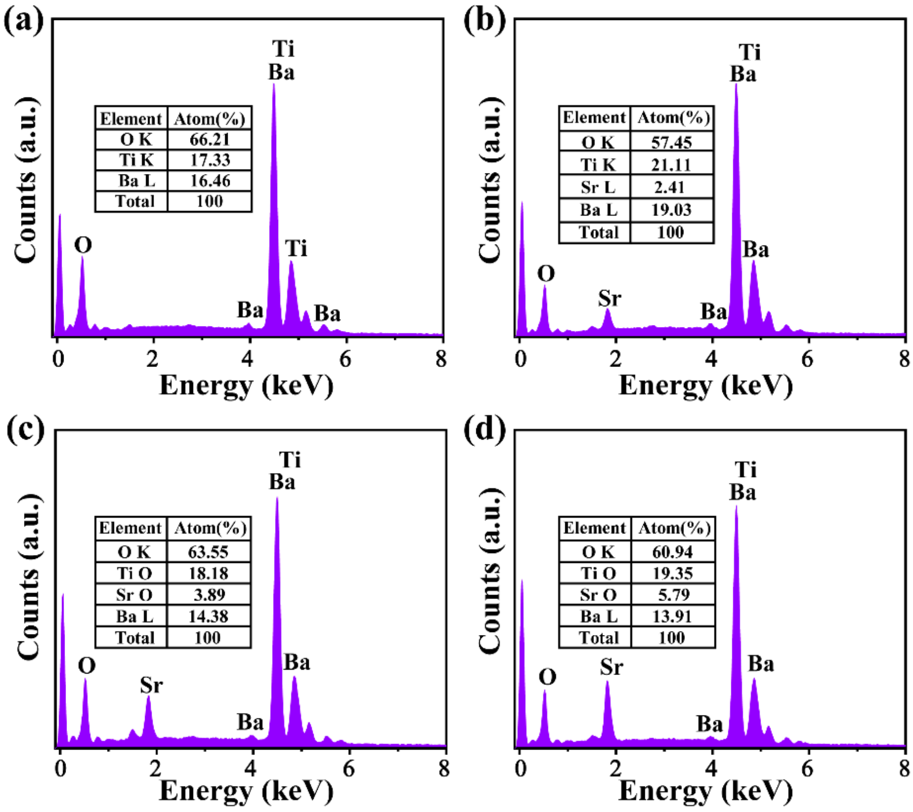

Figure S3. EDS patterns of $Ba_{1-x}Sr_xTiO_3$ nanopowders when x is: (**a**) x = 0; (**b**) x = 0.1; (**c**) x = 0.2; (**d**) x = 0.3.

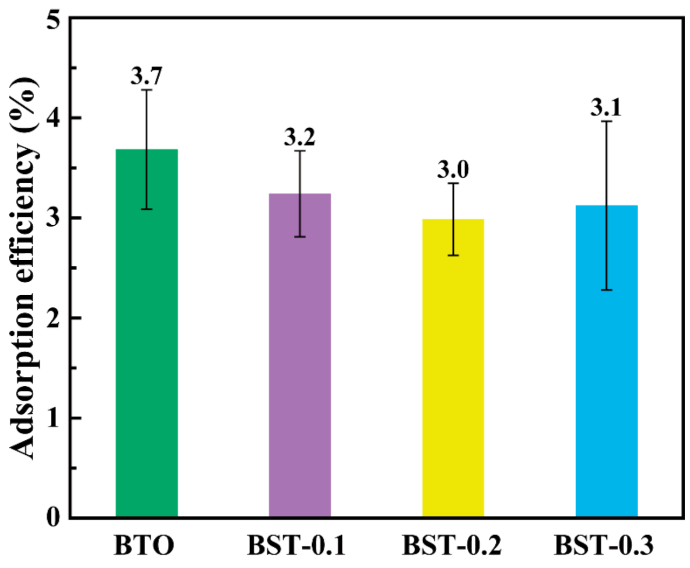

Figure S4. Adsorption efficiency of BST nanopowders on RhB solution within 30 min.

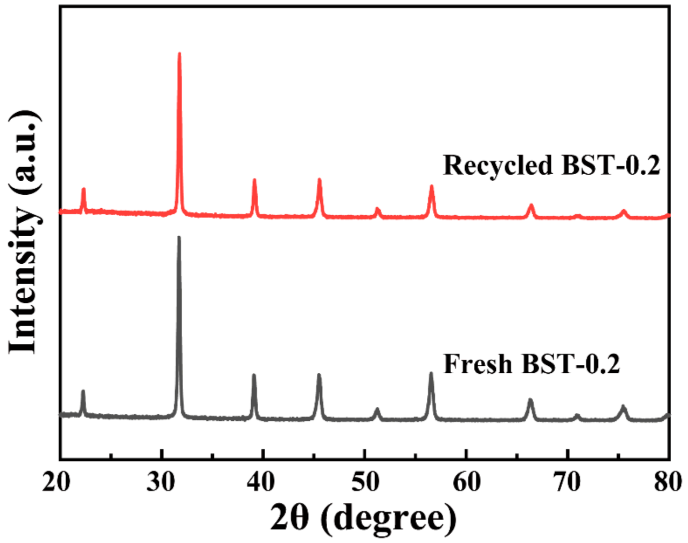

Figure S5. XRD patterns of BST-0.2 nanopowders before and after three cycles.

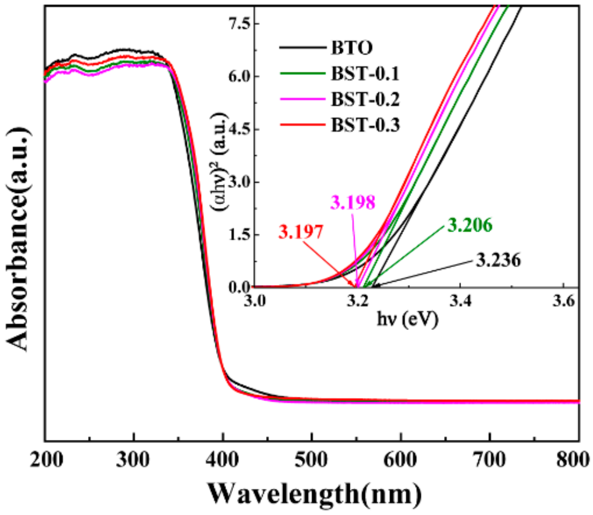

Figure S6. UV-Vis absorption spectrum of BST nanopowders.

Table S1. Comparison of the tribocatalytic performance with various materials.

Catalyst	Dye solution	Catalytic conditions	Degradation efficiency	Rate constant
ZnO nanorods [1]	RhB 5 mg/L	One PTFE magnetic bar, stirring speed of 1000 rpm	99.8% (60 h)	0.082 h^{-1}
$NiCo_2O_4$ DSNCs [2]	RhB 5 mg/L	One PTFE magnetic bar, stirring speed of 400 rpm	98.6% (56 h)	0.074 h^{-1}
Micron-sized poled BZT [3]	RhB 5 mg/L	One PTFE magnetic bar, stirring speed of 1200 rpm	99.6% (48 h)	0.077 h^{-1}
PVP-modified $Sr_{0.3}Ba_{0.7}TiO_3$ [4]	RhB 5 mg/L	Thermal cycling between 5 and 25°C with one PTFE magnetic bar	96.6% (12.5 h)	0.23 h^{-1}
STO nanofibers [5]	RhB 5 mg/L	One PTFE magnetic bar, stirring speed of 800 rpm	88.6% (24 h)	0.0877 h^{-1}
BST-0.2 nanopowders (This work)	RhB 5 mg/L	One PTFE magnetic bar in Glass beak, stirring speed of 300 rpm	88% (8 h)	0.2613 h^{-1}
		One PTFE magnetic bar in PTFE beak, stirring speed of 300rpm	99.1% (8 h)	—

Reference

1. Zhao, J.H.; Chen, L.; Luo, W.S.; Li, H.M.; Wu, Z.; Xu, Z.Y.; Zhang, Y.M.; Zhang, H.F.; Yuan, G.L.; Gao, J., Jia, Y.M. Strong tribo-catalysis of zinc oxide nanorods via triboelectrically-harvesting friction energy. *Ceram. Int.* **2020**, *46*, 25293-25298.
2. Ruan, L.J.; Jia, Y.M.; Guan, J.F.; Xue, B.; Huang, S.H.; Wang, Z.H.; Fu, Y.H., Wu, Z. Tribo-electro-catalytic dye degradation driven by mechanical friction using MOF-derived $NiCo_2O_4$ double-shelled nanocages. *J. Clean. Prod.* **2022**, *345*, 131060.
3. Tang, Q.; Zhu, M.D.; Zhang, H.F.; Gao, J.; Kwok, K.W.; Kong, L.B.; Jia, Y.M.; Liu, L.J., Peng, B.L. Enhanced tribocatalytic degradation of dye pollutants through governing the charge accumulations on the surface of ferroelectric barium zirconium titanate particles. *Nano Energy* **2022**, *100*, 107519.
4. Yang, B.; Chen, H.B.; Yang, Y.D.; Wang, L.; Bian, J.H.; Liu, Q.D., Lou, X.J. Insights into the tribo-/pyro-catalysis using Sr-doped $BaTiO_3$ ferroelectric nanocrystals for efficient water remediation. *Chem. Eng. J.* **2021**, *416*, 128986.
5. Cao, J.L.; Jia, Y.M.; Wan, X.M.; Li, B.B.; Zhang, Y.M.; Huang, S.H.; Yang, H.Y.; Yuan, G.L.; Li, G.R.; Cui, X.Z., Wu, Z. Strong tribocatalysis of strontium titanate nanofibers through harvesting friction energy for dye decomposition. *Ceram. Int.* **2022**, *48*, 9651-9657.
